# Supplementary material for: Extended ensemble simulations of a SARS-CoV-2 nsp1–5’-UTR complex
Source: PLoS Comput Biol. 2022 Jan 19;18(1):e1009804. doi: 10.1371/journal.pcbi.1009804 (PMC8803185; doi:10.1371/journal.pcbi.1009804)
Supplement: S1 Text — Text A: Convergence of the simulations. Text B: Characterstics of clusters. Text C: Details of clusters 2 and 3. Fig A: Survey for the clustering parameters. Fig B: Convergence of the secondary structure distribution. Fig C: Convergence of the hydrogen bond forming ratio. Fig D: Timecourse of the replica indices. Fig E: Distances to clusters in canonical simulations starting from the initial configuration. Fig F: Secondary structure distribution of nsp1 without SL1. Fig G: Representative structure of each cluster. Fig H: Surface area of interaction intefaces of nsp1. Fig I: Interactions between nsp1 and SL1 in cluster 2. Fig J: Interactions between nsp1 and SL1 in cluster 3. Fig K: Distances to clusters in canonical simulations starting from cluster 1 structures. Table A: Characteristics of the nsp1–40S ribosome complex models. Table B: Characteristics of SL1 binding regions of nsp1. Table C: Characteristics of each conformational cluster. (PDF) [file pcbi.1009804.s001.pdf]

Supporting Information for "Extended ensemble simulations of a SARS-CoV-2 nsp1–5'-UTR complex"

Shun Sakuraba*[1], Qilin Xie[2], Kota Kasahara[3], Junichi Iwakiri[4], and Hidetoshi Kono[1]

[1]Institute for Quantum Life Science, National Institutes for Quantum Science and Technology, Kizugawa, Japan
[2]Graduate School of Life Sciences, Ritsumeikan University, Kusatsu, Japan
[3]College of Life Sciences, Ritsumeikan University, Kusatsu, Japan
[4]Graduate School of Frontier Sciences, The University of Tokyo, Kashiwa, Japan

A Convergence of the simulations

We monitored the convergence of the simulation by two measures: the secondary structure and the hydrogen bond forming ratio. Fig B shows the probability distribution of the nsp1 secondary structure among different simulation lengths. We plotted the secondary structure forming probability using trajectories at 5–10 ns, 10–20 ns, 15–30 ns, 20–40 ns, and 25–50 ns of the simulation in replica 0. The secondary structure converged after 15–30 ns. We also tested the hydrogen bond forming ratio in Fig C. We took trajectories from x ns to $2x$ ns, e.g. 15 ns to 30 ns for the case of $x = 15$, and the probability of the hydrogen bonds between nsp1 and SL1 was assessed. Similar to the secondary structure, the hydrogen bond forming rates converged after $x \approx 15$ ns. From these results, we used 25–50 ns of the simulation in the subsequent analyses. We note, however, it is far from "fully converged" simulations expected in the rigorously performed extended ensemble runs; in Fig D, the index of Hamiltonians applied to 5 continuous trajectories are plotted. The result showed that the replica indices are bound by a rather narrow range and thus the full state mixing is unlikely to happen.

B Characterstics of clusters

Table B lists the residues involved in nsp1–SL1 binding. Fig G shows the structure of all 14 clusters identified in our analysis. Detailed characteristics of the clusters are listed in Table C and Fig H.

C Details of clusters 2 and 3

Cluster 2 interacted with SL1 via the interface regions (i), (ii), and (iv) (see Fig I). A remarkable feature of cluster 2 is the recognition of C19, C20, and C21. Arg43 and Lys47 in the region (ii) formed the salt-bridge with their backbone in 70.6 % and 100.0 % in cluster 2. The region (iv). The base of C21 was fliped out from the stem loop was stacked Asn126 amide group and Gly137 backbone. Asn126 also formed hydrogen bonds with C19 (96.7 %) and C20 (92.3 %).

Cluster 3 showed interactions with a wider range of SL1 than others. Lys11 and Lys125 frequently formed salt-bridges with U13 (91.5 %) and A14 (88.8 %), respectively. His134 showed a high contact frequency with C32. In addition, the side chain of Asp126 entered the center of the stem loop and formed the hydrogen bonds with the bases of U17 and C20.

*sakuraba.shun@qst.go.jp

References

[1] David L Davies and Donald W Bouldin. A cluster separation measure. *IEEE transactions on pattern analysis and machine intelligence*, 1(2):224–227, 1979.

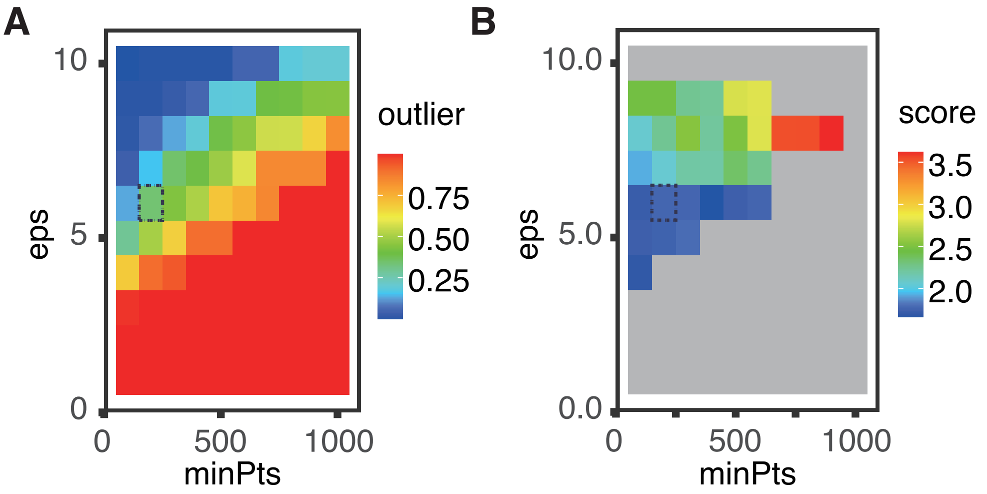

Figure A: Survey for the clustering parameters. The DBSCAN clustering method was applied with 64 parameter sets, which are the combination of eight eps and eight minPts parameters. For each parameter set, (A) ratio of outliers and (B) Davies-Bouldin index [1] were assessed. Davies-Bouldin index is a metric to evaluate results of cluster analyses; lower values indicate better results. The regions filled in gray in the panel (B) indicate the conditions that the number of clusters was zero or unity. The distribution indicates that lower eps values yield better results of clustering. At the same time, lower eps and higher minPts increase the ratio of outliers. Clustering with eps = 6 and minPts = 200, marked as the dashed rectangle, yielded 14 clusters with 37.5 % of outliers. We discussed the clusters based on this condition.

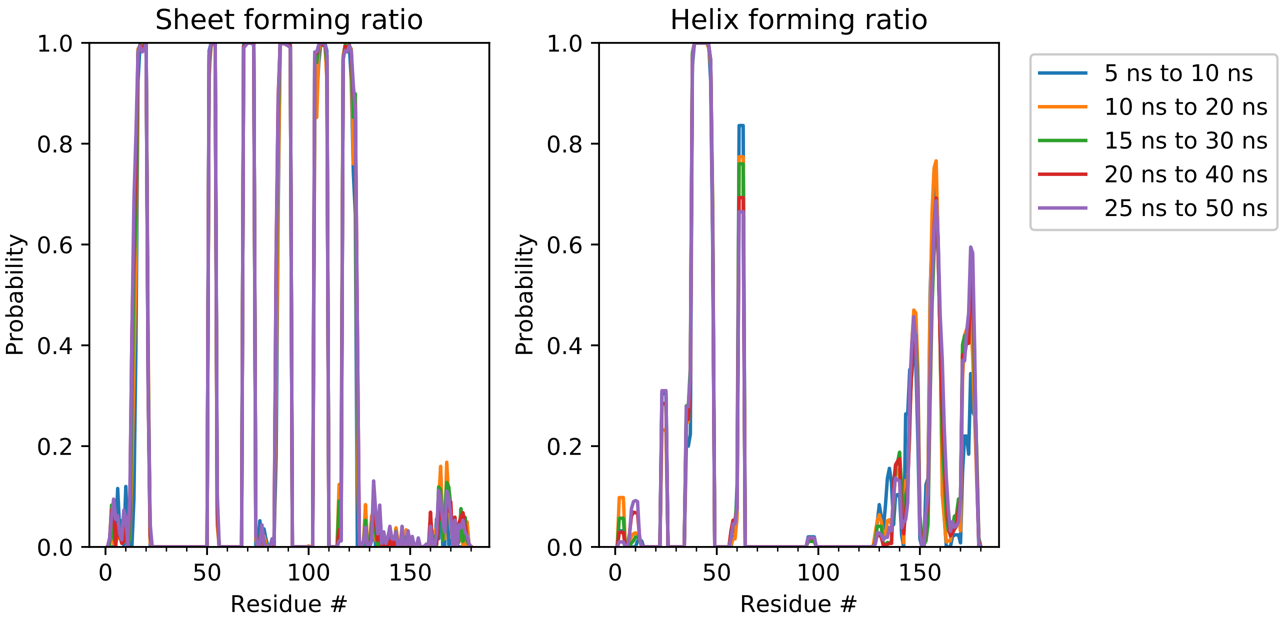

Figure B: Secondary structure distribution of the simulation corresponding to replica number 0. “Helix” includes the DSSP assignment of α-helix, 3-10 helix and π-helix. “Sheet” includes both β-bridge and β-ladder.

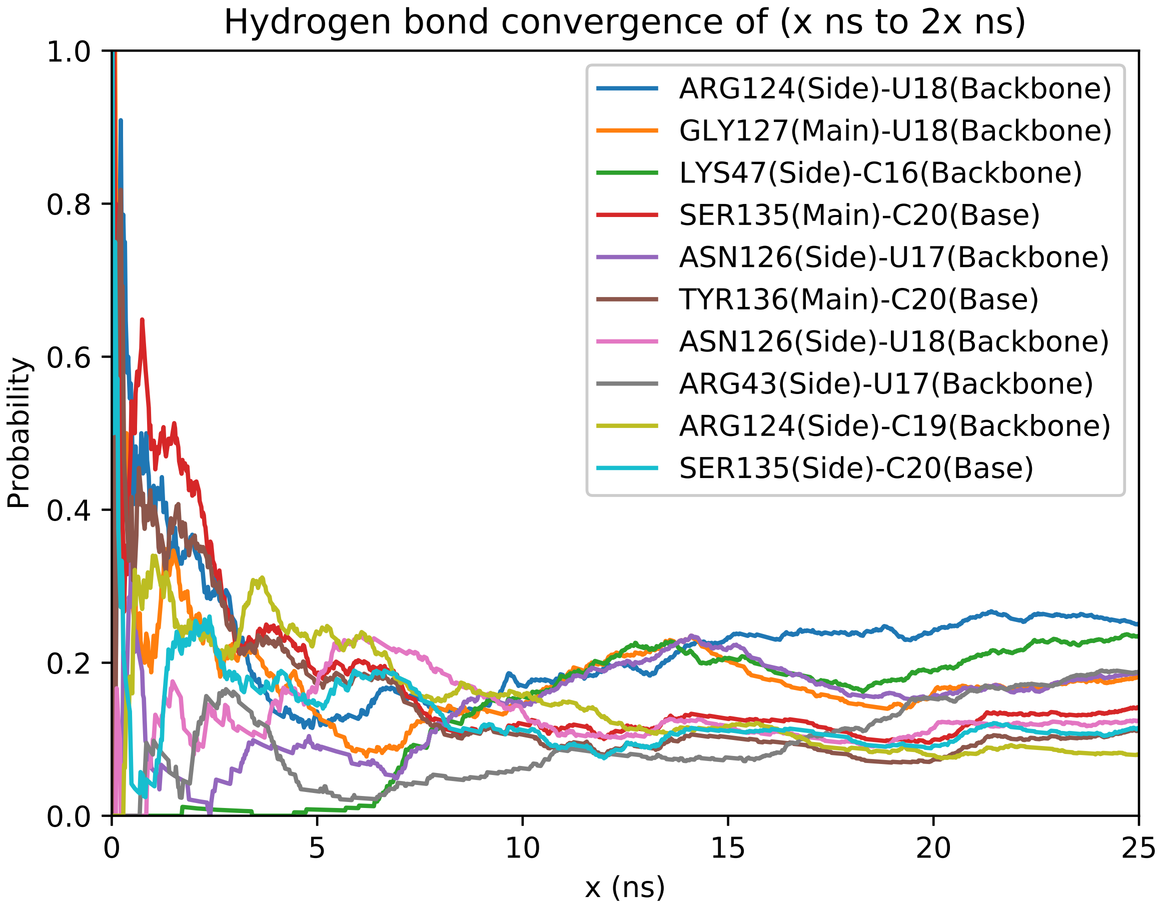

Figure C: Hydrogen bond forming ratio between x ns and $2x$ ns of the simulation in replica 0. Hydrogen bonds having the 10 largest probabilities among 0–50 ns simulation were selected and plotted in the figure.

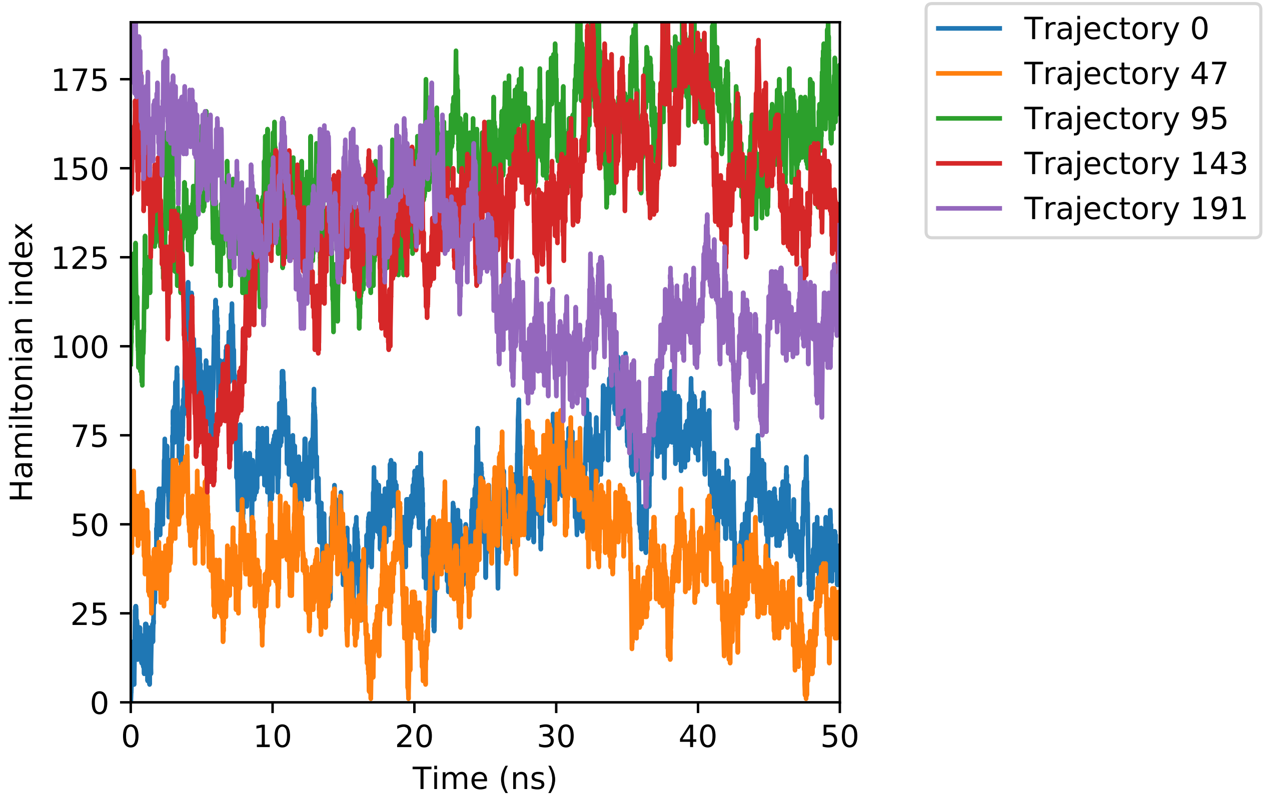

Figure D: Timecourse of the replica indices for the nsp1-SL1 simulation. Five continuous trajectories were chosen whose initial Hamiltonian indices were 0, 47, 95, 143 and 191. The indices of the Hamiltonian of these replica were plotted along the time. Note replica 0 corresponds to the “lowest temperature”, while replica 191 corresponds to the ”highest temperature”.

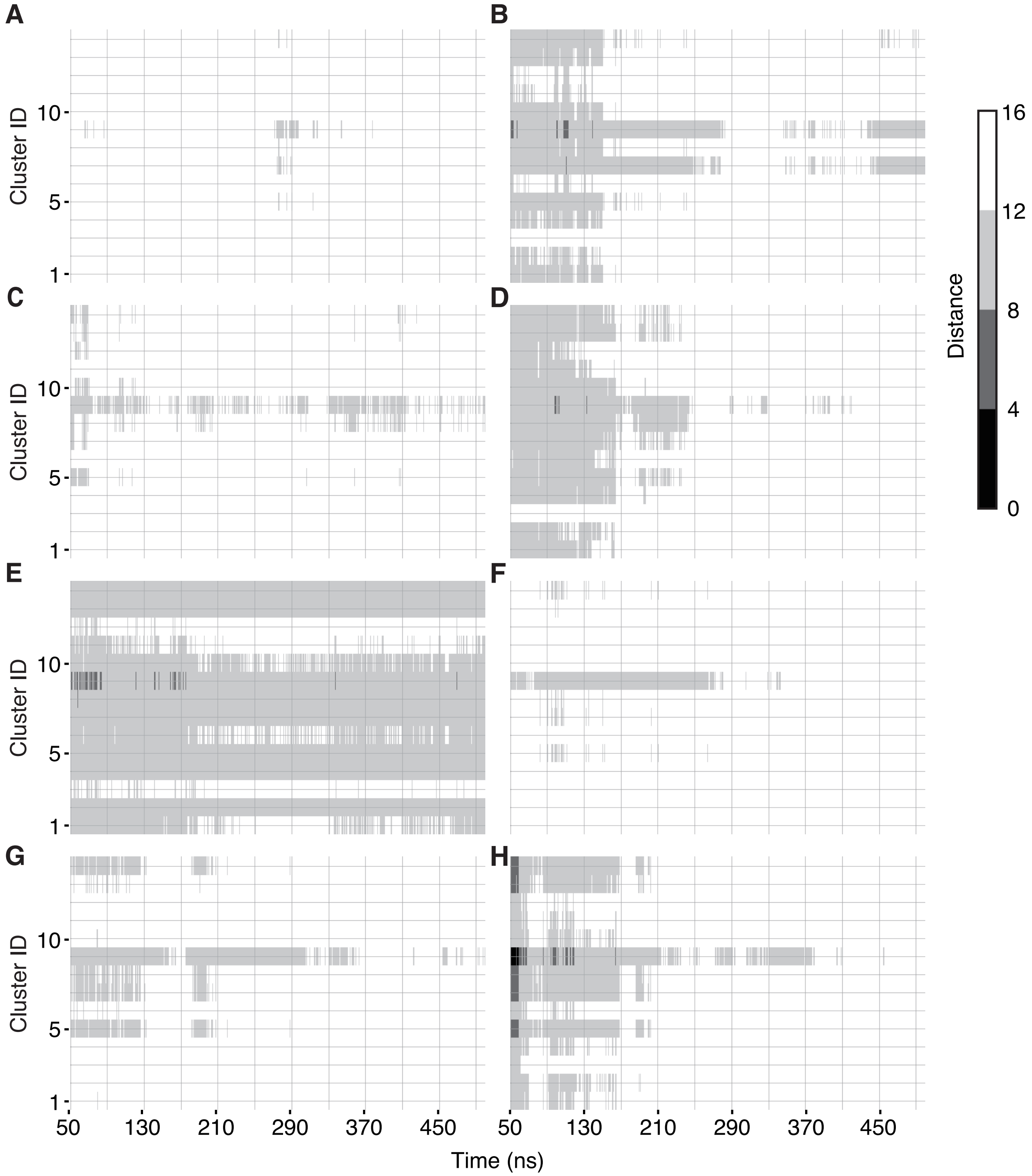

Figure E: Distances to each cluster from each snapshot in the canonical simulations started from the initial structure of the REST2 simulation. Trajectories for the eight simulations with different initial atomic velocities were shown in (A) through (H). The distance was defined as the Euclidian distance of the nsp1–SL1 contact matrixes between a cluster center and a snapshot. Any 500-ns trajectory of the canonical simulations did not converge to any cluster observed in the REST2 simulation.

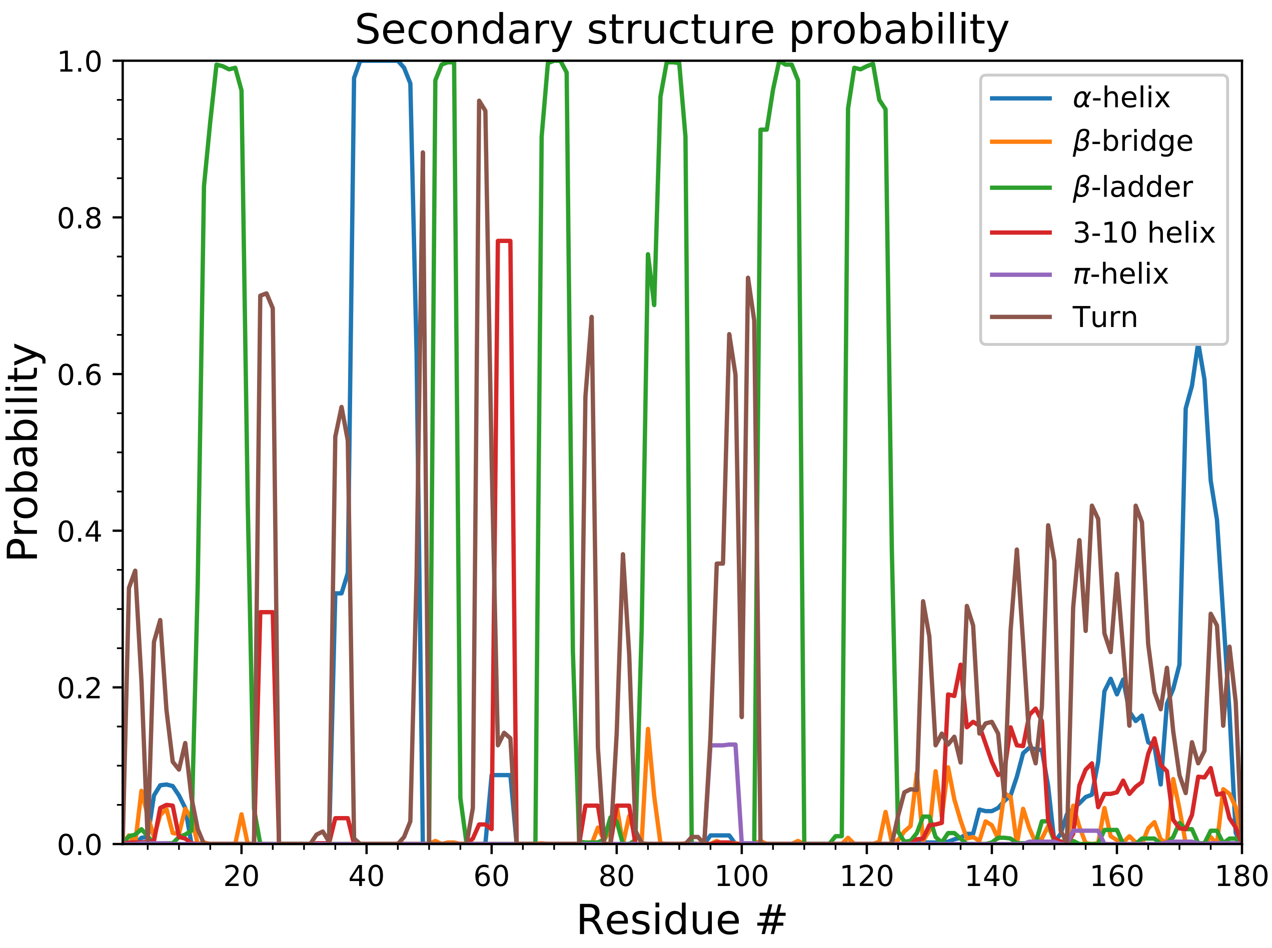

Figure F: The secondary structure distribution of nsp1 without SL1.

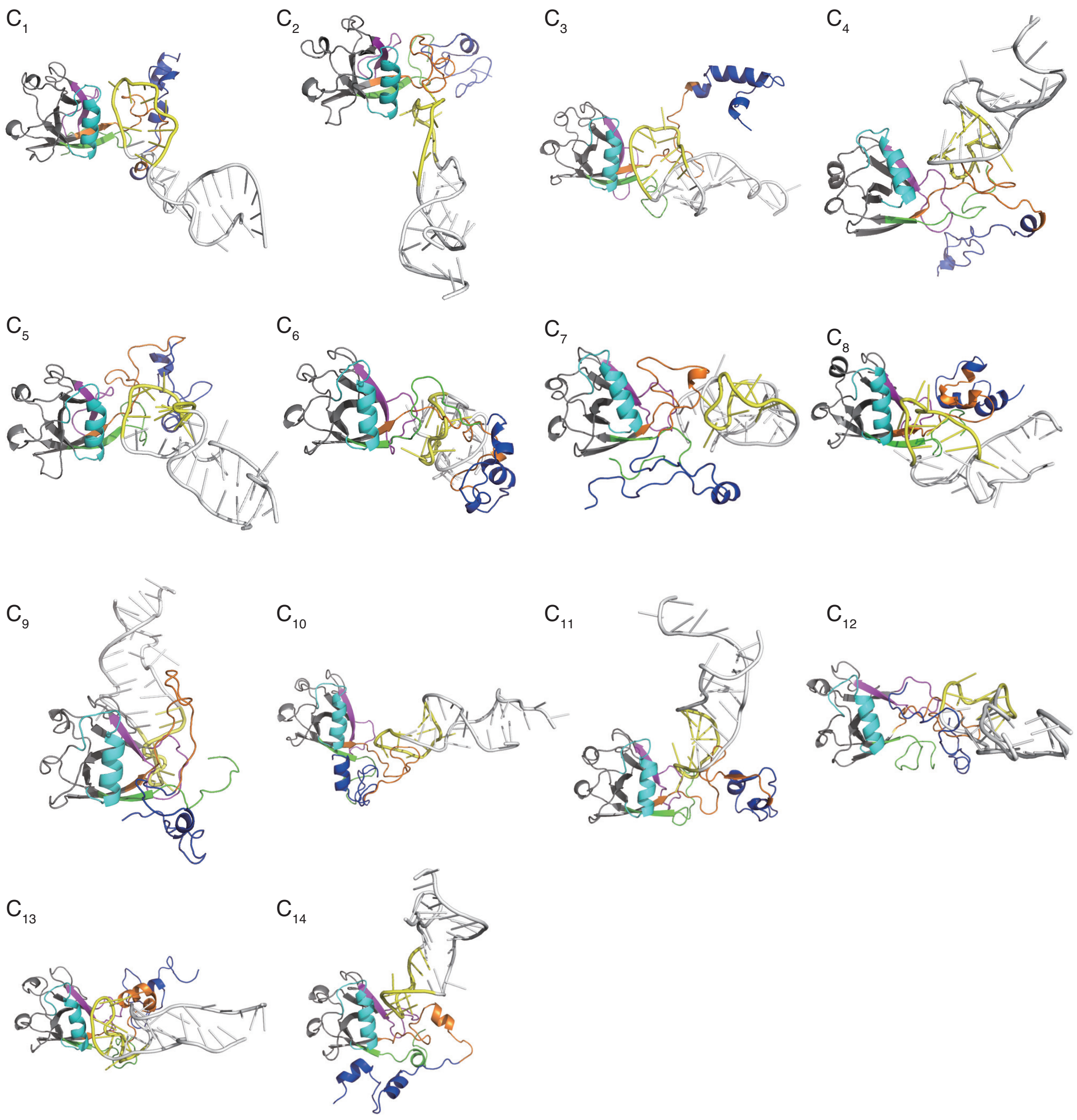

Figure G: Examples of a snapshot in each cluster. The panels (C_1) through (C_{14}) show snapshots of clusters 1 through 14, respectively. The interface regions (i) through (v) are shown in green, cyan, magenta, orange, and blue ribbons. The 16–26th bases of SL1 are shown in yellow.

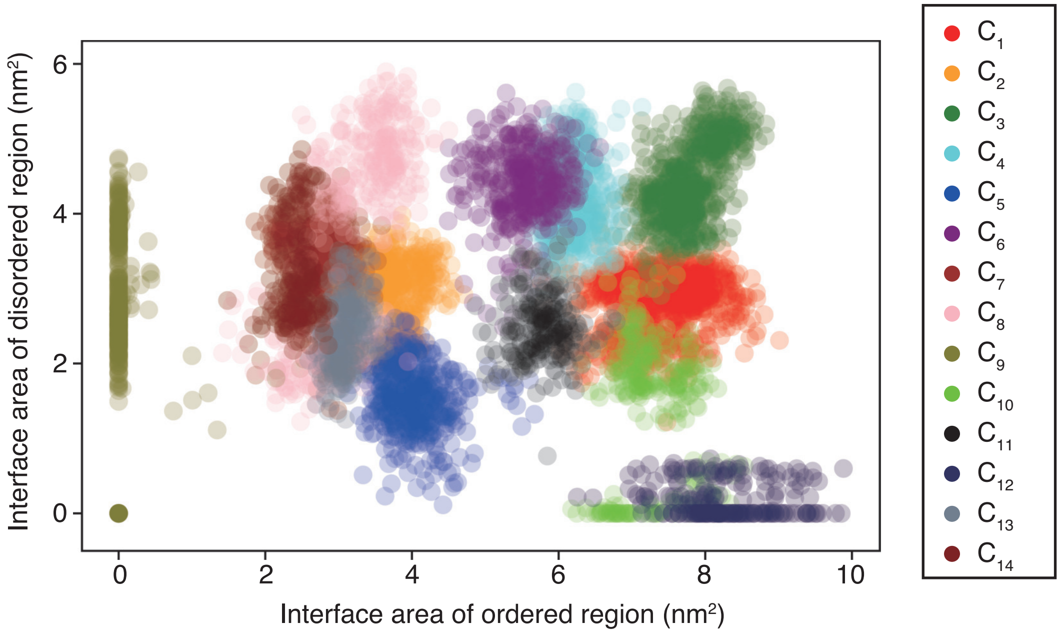

Figure H: The surface area of interaction interfaces of Nsp1 in each snapshot. C_1 through C_{14} represent clusters numbered 1 to 14, respectively. The horizontal and vertical axes indicate the area on ordered and disordered regions, respectively. Color corresponds to the cluster ID.

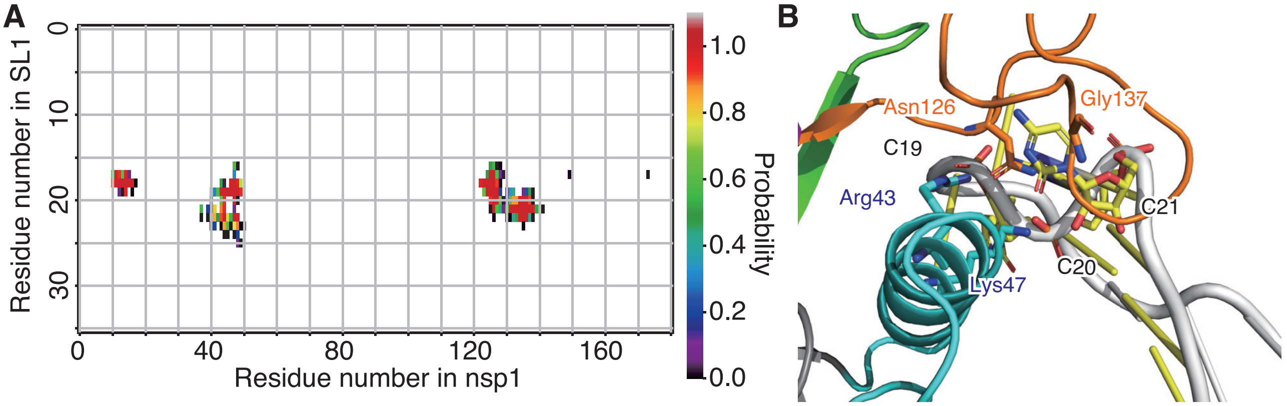

Figure I: Interactions between Nsp1 and SL1 in cluster 2. (A) Pairwise contact probability for each cluster. See the legend of Fig. 4 in the main paper. (B) A snapshot in cluster 2.

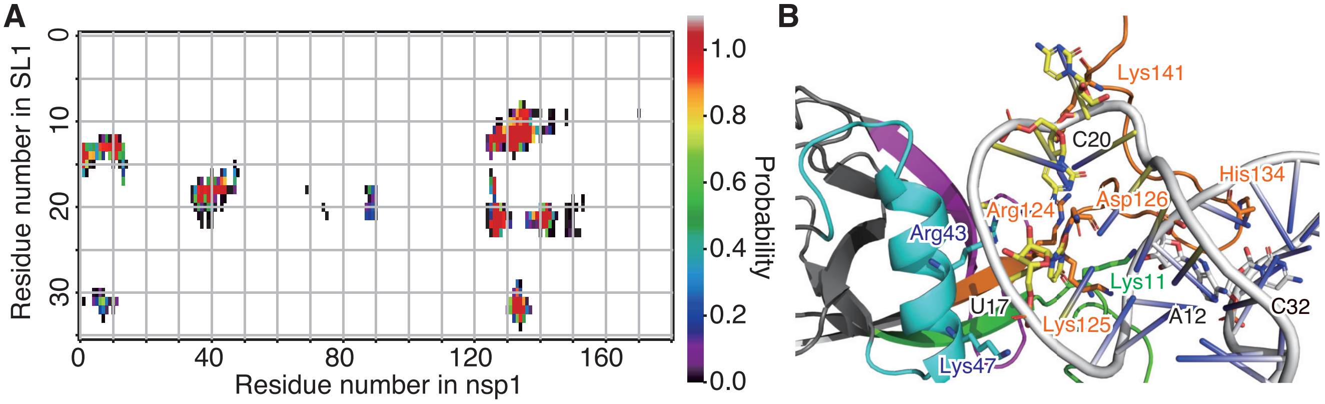

Figure J: Interactions between Nsp1 and SL1 in cluster 3. (A) Pairwise contact probability for each cluster. See the legend of Fig. 4 in the main paper. (B) A snapshot in cluster 3.

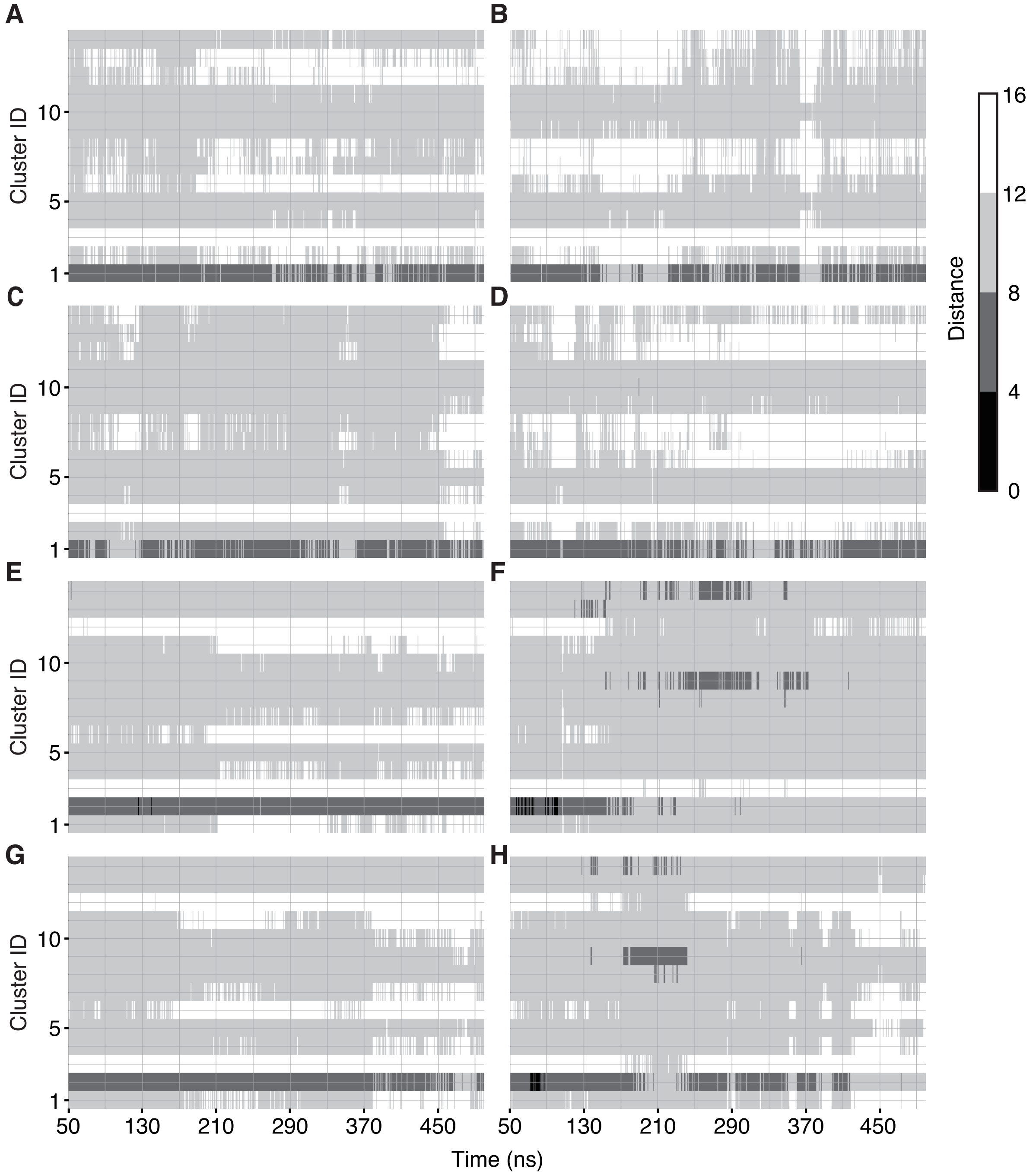

Figure K: Distances to each cluster from each snapshot in the canonical simulations started from the cluster 1 (A, B, C, and D) and cluster 2 (E, F, G, and H). See also the legend of Fig E. All the trajectories of the simulations started from a conformation in the cluster 1 (A, B, C, and D) were trapped into the cluster 1 during the simulation time. In the case of simulations started from a conformation in the cluster 2, two runs stably kept the conformation of the cluster 2 (E and G) while in the other two runs conformational changes to a few specific clusters were observed (F and H).

Model no.	Distance (Å)	Collisions
1	28.8	6
2	**8.3**	**4**
3	30.7	9
4	**19.7**	10
5	**16.1**	25
6	26.9	9

Table A: Characteristics of 6 candidate model structures of the nsp1-40S ribosome complex. Bold texts indicate either the distance (between nsp1 K120 and S3 K62) is within the cutoff ($<$ 25 Å) or the smallest number of collisions among the candidates.

Region ID	Residues	%*
(i)	1–18	72.1
(ii)	31–50	81.8
(iii)	74–90	63.2
(iv)	121–146	97.4
(v)	147–180	59.2

* The probability to form at least one contact between the region and SL1.

Table B: Characterization of SL1 binding regions of Nsp1.

Cluster ID	Total%	(i)%	(ii)%	(iii)%	(iv)%	(v)%	Representative contacts
1	15.5	14.0	100	100	100	98.5	Arg124–U17
2	9.88	100	100	0	100	1.77	Lys47–C20
3	7.42	100	100	81.4	100	6.96	Arg126–C20
4	4.98	100	100	100	100	0	His134–C20
5	4.91	37.5	100	91.0	100	100	Gly127–U18
6	4.88	100	0	100	100	80.6	Arg77–C20
7	3.98	100	0	100	100	0.91	Ser142–A22
8	3.60	100	100	24.3	100	8.16	Arg43–C19
9	2.62	13.0	0.88	87.8	0.88	0	Arg73–A22
10	2.30	52.7	77.5	86.0	100	100	Asn162-U18
11	1.85	100	100	100	100	13.8	Ser135–C20
12	1.36	0	0	34.0	100	100	Ser135–A22
13	1.31	75.5	100	45.7	100	0	Gly127–C19
14	1.20	0	100	100	100	1.74	Arg43–C20
outliers	34.2	84.6	85.7	41.0	100	88.1	-

Table C: Characteristics of each conformational cluster.

Detected interactions	%	Bond type
Arg124–U17	96.0	H-bond
Asp75–U18	95.2	H-bond
Ser40–U17	84.3	H-bond
Ala131–C19	78.8	H-bond
Ser135–C16	78.4	H-bond
Arg124–U18	72.0	H-bond
Lys47–C16	71.2	H-bond
Asn126–C16	59.0	H-bond
Arg43–U17	56.4	H-bond
Lys47–C16	82.3	salt-bridge
Arg43–U17	81.1	salt-bridge
Arg43–U18	76.2	salt-bridge
Arg124–U17	75.2	salt-bridge
Lys141–A14	57.4	salt-bridge

Table D: Hydrogen bonds and salt-bridges in cluster 1.

Detected bonds	%	Bond type
Lys47–C20	96.9	hbond
Asn126–C19	96.7	hbond
Asn126–C20	92.3	hbond
Lys47–U18	82.3	hbond
Arg43–C20	69.6	hbond
Ser40–C21	63.8	hbond
Gln44–C20	55.5	hbond
Lys047–C20	100.0	salt-bridge
Arg043–C20	70.6	salt-bridge

Table E: Hydrogen bonds and salt-bridges in cluster 2.

Detected bonds	%	Bond type
Asn126–C20	96.5	hbond
Asn126–U17	87.0	hbond
Lys141–C21	85.8	hbond
Lys125–A14	77.3	hbond
Lys11–U13	77.1	hbond
Ser142–A22	72.8	hbond
Met1–C15	70.7	hbond
Ser135–U11	65.2	hbond
Ser40–U18	56.2	hbond
Asn126–G23	55.6	hbond
Lys011–U13	91.5	salt-bridge
Lys125–A14	88.8	salt-bridge

Table F: Hydrogen bonds and salt-bridges in cluster 3.
