# Supplementary material for: Extended ensemble simulations of a SARS-CoV-2 nsp1–5’-UTR complex
Source: PLoS Comput Biol. 2022 Jan 19;18(1):e1009804. doi: 10.1371/journal.pcbi.1009804 (PMC8803185; doi:10.1371/journal.pcbi.1009804)
Supplement: Attachment — Submitted filename: response_rev1.pdf [file pcbi.1009804.s004.pdf]

We thank both reviewers for carefully reading the manuscripts and giving us many constructive and detailed comments. Our responses to the individual comments are presented below.

Reviewer #1: Sakuraba et al present a very well written study about the mechanisms by which SARS-CoV2's cognate RNA evades the translation shutoff operated by viral protein nsp1 interacting with the host ribosome. Using enhanced sampling molecular dynamics simulations, they investigate the specific interaction between nsp1 and the SL1 region of the RNA. Needless to say, research on SARS-CoV2 is very valuable and timely. However, working on SARS-CoV2 implies both high rewards and high risks: the competition on this topic is fierce, and advances are published almost daily, rendering some papers "obsolete" even before they can be published. This is typically the case here.

The main issue is the the absence of the ribosome in the simulations: interactions between nsp1 and the ribosome are almost certain to change the conformation and/or conformational dynamics of nsp1, especially considering its partly unstructured nature, and no evidence exists that nsp1 binds the viral RNA in the absence of the ribosome ("Whether SARS-CoV-2 nsp1 and SL1 bind without the ribosome is controversial."). The authors are aware of this and very clearly state it in the "limitations of this study" section. It is unfortunate for the authors that the structure of the ribosome/nsp1 complex was published after their simulations had been performed and before this work could be published, but this does not change the fact that the mechanisms that the authors are exploring are, at best, tentative, and at worst, nonexistent.

To alleviate this, the authors should try to incorporate information from the nsp1/ribosome structure into their simulations and compare with their current results. This could be done for example by running additional REST2 simulations (possibly with less replicas than the original simulations) in which the ribosome-bound conformation of nsp1 is imposed using restraints, or by evaluating the difference in free energy between both states of nsp1... Thus providing information about the role of the ribosome in the recognition mechanism between nsp1 and SL1 would be quite valuable.

We admit that current result of nsp1-SL1 simulation is still miles away from suppressing the SARS-CoV-2 proliferation and we deeply thank the reviewer for understanding the situation behind this manuscript. As we discussed in the initial version of the manuscript, the simulation of nsp1-SL1-ribosome 40*S* complex will be necessary to encompassing the nsp1 study further. However, we still consider that the current simulation results of nsp1-SL1 system adds information for nsp1 research in the following ways:

1. The binding position of SL1 found in the simulation was consistent to the current experimental (ambiguous) structure of ribosome-nsp1 complex.

2. The residues involved in the binding also matched with the experimentally known mutation that loses the binding ability.

3. The result also implies the importance of ribosomal protein RS10's disordered region at its C-terminal.

Including these findings, we revised the manuscript in the following ways. First, we added a new section in which we explain how the model of the ribosome-nsp1 structure is constructed based on updated experimental information.

> *(Page 6, line 203) To compare the binding poses obtained from the simulation with the recent experimental results, we modeled the complex structure of nsp1 and 40S ribosome based on the density map from the cryo-EM and the cross-linking experiment. ......*

Additionally, we added a new section in "Results and Discussion" named "SL1's binding position in 40S ribosome-nsp1 complex", discussing the connection between the simulation finding and the current model.

> *(Page 9, line 329) We further investigated the consistency between the known structure and the SL1's binding preference. For that purpose, we constructed the model of the 40S ribosome-nsp1 complex. ...*

On the other hand, due to the point 3 we discussed above, we currently consider that the simulation of nsp1-SL1-ribosome complex is quite difficult to achieve, thus we would like to leave this for a future study. We updated the comment in the section "Limitation of this study".

> *(Page 11, line 442) Our simulation was performed without the ribosome. ... Furthermore, with the 40S ribosome, ribosomal proteins S3 and S10 as well as rRNA hairpins at around residue 540 may interact with nsp1 or the SL1 as presented in Fig. 8, which makes a proper sampling of the configurations difficult. ...*

We believe these changes make this study's contribution clearer.

> *In any case, until additional simulations are performed, the paragraph "Distance between the nsp1 N-terminal domain and C-terminal helices" is much too tentative and should be removed. In my opinion, the assumption "This indicates that the configuration observed in the cryo-EM structure, which does not include SL1, is unlikely to happen when nsp1 is complexed with the SL1" cannot be safely made when one side of the comparison is done with the ribosome and the other one without.*

We performed the simulation without SL1 (nsp1 only) to confirm our speculation that SL1 binding decreases the distance between the nsp1 N-terminal domain and

C-terminal helices in the initial version of the manuscript. Indeed, the simulation with nsp1 only resulted in shorter nsp1 N-C domain distance and thus we removed the speculation in the original manuscript. We revised the manuscript thoroughly based on the simulation results.

> *(Page 3, line 93) After preparing the system we also prepared the system without SL1 by removing it from the nsp1-SL1 initial structure (the total number of atoms was 223,598).*

We also added the comparison between nsp1 with and without SL1 as a separate section.

> *(Page 8, line 301) Nsp1's globular region and IDR do not stably interact with each other*
>
> *Next, we investigated the intra-residue contacts within nsp1 with and without SL1. Figure 6 shows the contact map between nsp1 residues. The result showed that nsp1's globular region and IDR did not have stable contacts regardless of the presence of the SL1. ...*
>
> *(Page 8, line 317) Nsp1's IDR may be aligned with SL1*
>
> *Although there were no stable contacts between nsp1 globular region and IDR, the radius of gyration Rg of the nsp1 without SL1 was smaller compared to that without SL1 (Fig. 7A), suggesting that nsp1 alone is more compact compared to nsp1 with SL1. ...*

> *Another important issue is the use of constraints on the RNA. In the "limitations" section, the authors claim that they are not really needed and thus do not impact the results meaningfully. But then, why were they used? What is the point of having SL1 in the "hot" region of the REST2 method if it is restrained? Figure 1C shows that no specific conformation emerges from the simulations for SL1; how is this compatible with the use of restraints, and what would happen without them? The authors should clarify this better than what is currently done in "limitations", and/or remove the restraints altogether in the additional simulations suggested above.*

Sampling configurational space of RNA is generally considered difficult even if the force field problems are set aside. Thus, we decided to narrow the configuration space of RNA, as stacking G-C Watson-Crick pairs are generally considered strong, and experimental evidence suggested that breaking G-C pairs leads to the failure to recognize the SL1. However, we included RNA into the REST2 region because we wanted to weaken interactions between RNA-protein as well as RNA-ions to sample proper configurations of the Protein-RNA complexes. To explain underlying reasons, we revised the manuscript:

(Page 4, line 107) Therefore, even though some base pairs of SL1 were restrained, the interactions between nsp1 and SL1 as well as SL1 and solvent were scaled in the REST2 simulation, allowing broader configurations to be sampled.

Another possible issue (this time not mentioned by the authors) relates to starting the MD simulations from an unbound state of the nsp1/RNA complex. Considering the length of the IDR in its extended conformation, differences in the initial positioning of the RNA along the IDR could result in different locations for the first contact between partners, conditioning further binding events (in particular the partial folding of the bound IDR that the authors observe). Yet the representativity of the binding simulation is not discussed by the authors at all...

We are considering that the problem of the initial configuration was mitigated by the extended ensemble simulations (REST2). We comment this issue combined with the questions raised by the reviewer #2.

A few more minor points:

- DBSCAN clustering: silhouette and/or Davies-Bouldin scores should be provided to assess the meaningfulness of the clustering and to justify the choice of the clustering hyperparameters;

We updated the supporting information for the choice of hyperparameters. (Supporting information Text 1, Fig. SF1)

- The right panel in figure 3 is not very informative: it is zoomed out too much, making the binding mode of nsp1 hard to grasp. I would suggest replacing the left panel by the right panel, and replacing the right panel by a zoomed-in view centered on the nsp1 binding site.

We withdrew this model figure (because we removed the nsp1 N-C domain distance) and reflected the comment to the new figure about nsp1-ribosome complex structure.

To conclude, reviewing this work is a bit of a conundrum for me. On the one hand, the article is well-written, the methods used are state-of-the-art, and the authors are quite straightforward about the limitations; on the other hand, it is debatable whether or not the results advance our actual understanding of SARS-CoV2's infection machinery. I hope additional simulations can be carried out, and major revisions to the article performed,

to fortify the article's findings and make it suitable for publication in PLoS CB.

Thank again the reviewer for constructive comments. We hope that the additional modeling as well as additional simulations (some are also added in response to reviewer 2) makes our contribution clearer.

Reviewer #2: The work investigates, by MD simulations, a challenging system, SARS-Cov-2 nsp1 - 5'-UTR protein-RNA complex.

The paper is based on one REST2 enhanced sampling method atomistic simulation with impressive 192 replicas on one side but quite short 50 ns simulation time per replica on the other, resulting in 9.6 microsecond total time, which is not bad considering the size of the system. The currently recommended first-choice versions of the AMBER force field are used. Due to the absence of relevant structural data, preparation of the starting structures was very challenging. The authors have used a relevant (considering the circumstances) protocol. In summary, the work deserves publication. However, substantial revisions are needed before it can be published in order to better understand the significance/limitations of the computations, to make the paper more understandable for non-experts and to avoid overstating/over-interpretations.

Obviously, considering the inevitable approximations in the preparation of the starting structure, known deficiencies of both the protein and RNA force fields (which often struggle with even much simpler and better defined systems) and the obvious sampling limitations, a quantitative accuracy of simulations of the present system is not achievable. This of course does not preclude the publication, as I noted above. However, I request the authors moderate their tone, rather telling that the modelling "suggests certain things" than "reveals things". The paper actually already contains a useful paragraph discussing limitations of the simulations, but the discussion should be extended by noting well-known (albeit often ignored) problems that can complicate simulations of protein-RNA complexes (https://wires.onlinelibrary.wiley.com/doi/10.1002/wrna.1405, https://pubs.acs.org/doi/10.1021/acs.chemrev.7b00427) and also sampling limitations which I assume are severe; see below.

The moderation would not undermine the paper while the reader would have a more balanced information. The credibility of the paper may actually be improved. There are currently many trashy MD papers in the literature which to my opinion degrade the MD simulation field. So, the authors may

actually profit from distancing their work from that part of the literature, as their calculations per se are interesting.

We totally agree to the reviewer's comment. As the reviewer suggested, we revised the manuscript to prevent overstatement and tried to moderate the current finding from the research, especially in the abstract and conclusion.

(Page 1, Abstract) ... Simulation results suggested that the stem loop structure of the SARS-CoV-2 RNA 5'-untranslated region (SL1) binds to both nsp1's N-terminal globular region and intrinsically disordered region. ...

(Page 1, Author summary) ... Our simulation results suggest that the binding between nsp1 and SL1 is multi-modal. The results also imply that both the N-terminal globular part and the C-terminal flexible tail of nsp1 are involved in the binding. ...

(Page 2, line 31) In the present research, therefore, our aim was to accumulate information about the molecular mechanism by which SARS-CoV-2 RNA evades nsp1. As a first step, we focused on how and where SARS-CoV-2 5'-UTR binds to nsp1, and tackled the problem from computational simulations. ...

(Page 13, line 490) Upon the binding, the disordered region may extend along the stem loop. The interaction analysis further suggested that the hairpin loop structure of the 5'-UTR stem loop binds to the N-terminal domain and the intrinsically disordered region of nsp1. ...

The supplementary information presents very short discussion of the convergence of the REST2 simulation based on secondary structure and H-bond rate in the basic replica 0. To the best of my knowledge, it is not reliable analysis of a convergence of RE simulation so the claim of convergence is deceptive.

To analyze convergence would require to first monitor how do the continuous replicas travel in the replica space, which should be documented, and in addition convergence would have to be proven in the space of all continuous replicas. I do not think the simulations are converged, considering the complexity of the system. For such a system 50 ns RE simulation, even with 192 replicas, is insufficient to simulate converged binding. While more replicas may help reduce trapping, they reduce the time accumulated in the unbiased replica. Such simulations can maximally show some encounter complexes but would not reveal, to my opinion, true binding events in case the binding requires any significant induced fit mechanism for which the molecules would need to diffuse through the conformational space. For methods such as REST2 I can imagine realistically an order of magnitude speed up compared to plain simulations,

but not more. Note also that efficiency of the RE methods is highly system-dependent and may sometimes be even compromised when higher-order replicas excessively increase the sampled space, when entropy and diffusive barriers are present etc. The folding/binding events are in reality finite-time single molecule events/micro-pathways which may prevent their realization in RE simulations (which obscure kinetics) for systems requiring more physical time to traverse the conformational space.

As the reviewer commented, we checked the trajectory of the replica states and was indeed very problematic. We revised the manuscript explicitly explaining the limitation of the current simulation research (details are described in the response to the next comment).

(Reviewer #1)

Another possible issue (this time not mentioned by the authors) relates to starting the MD simulations from an unbound state of the nsp1/RNA complex. Considering the length of the IDR in its extended conformation, differences in the initial positioning of the RNA along the IDR could result in different locations for the first contact between partners, conditioning further binding events (in particular the partial folding of the bound IDR that the authors observe). Yet the representativity of the binding simulation is not discussed by the authors at all...

(Reviewer #2)

Enhanced sampling is not a panacea and rigorous analyses of performance often reveal problems https://doi.org/10.1146/annurev-biophys-042910-155255 Based on the presented data I have absolutely no idea about the convergence and I would assume that the REST2 run shows rather some mixture of encounter complexes accessible from the starting structure, not necessarily the ultimate binding. Thus, the section discussing limitations should be considerably broadened and the authors need to either monitor convergence by some valid tools or clearly admit that they did not monitor convergence at all. Some examples of papers with similar methods where convergence is better analyzed are for example: https://rnajournal.cshlp.org/content/21/9/1578.long, https://pubs.acs.org/doi/pdf/10.1021/acs.jctc.8b00955, https://www.nature.com/articles/s41467-021-21105-7; there are certainly also other works where convergence of RE simulations is analyzed rigorously. I emphasize that I do not expect the authors to prove the convergence of their ensemble as that is likely far beyond the currently available simulation techniques and hardware. Some degree of convergence can be achieved for fast folding proteins or very small nucleic acids system, or for mutual binding of essentially semi-rigid systems (say two folded proteins), but not for this system. However, discussion of the limitation and

perhaps some insight how extended the sampling is would be essential to make the work understandable.

Without this the analysis is rather hand-waving. I would tend to assume that the REST2 run reflects rather pre-binding of the complex within the approximation of the force field and assuming the start. Key parts of the binding landscape might still remain unvisited.

I would also suggest that authors briefly explain basics of the REST2 method and how it compares with other enhanced sampling methods. No mathematics is needed, just some plain explanation for non-experts, perhaps using SI if more space is needed. Presently the paper is a common MD simulation text with lot of technical jargon which is understandable for specialists but I do not think experimental researchers would grasp the computations. It is also one of the reasons why the impact of atomistic MD studies of protein-RNA complexes in the literature outside the internal MD community is not large. Other researchers often do not understand the papers and do not trust the methods due to notorious overstatements, over-interpretations and excessive self-confidence. Actually, could you please explain better which points exactly are used for clustering, what exactly is in the matrix?

To improve the paper, for comparison, it might be very useful to add to the paper a moderate set of standard simulations, first to see what happens when initiating from the starting structure in standard simulations and then perhaps initiating some simulations from interesting candidate structures detected in REST2. To see, if they settle down in some metastable conformations.

We deeply thank both reviewers for the constructive comments. We answer the questions from both reviewers in this response. In the revised manuscript we added following simulations and analyses, and we tried to carefully explain the ideas to non-experts without being overstatement:

1. We performed a set of standard simulations from the initial model of the nsp1-SL1 and discussed the structures obtained therein. Starting from the initial model, we performed 8 standard MD simulations for 500 ns long. The simulations support that the trajectories obtained from the initial configuration with the conventional MD are different from the extended ensemble results, supporting that the extended ensemble simulation at least prevented the simulation to be trapped into a configuration close to the initial configuration.

(Page 4, line 130) In addition to these extended ensemble simulations, we performed 8 MD simulations of 500 ns length each, starting from the initial configuration of the nsp1-SL1 complex to see the difference between conventional simulations and extended ensemble simulations. First 50 ns chunks of the simulations were removed from the data as the equilibration

time, and remaining 450 ns simulation results were used in the subsequent analyses.

2. We also performed another set of 8 conventional MD simulations starting from the clusters 1 and 2. In 6 out of 8 cases the simulation remained stable, supporting that bound structures found in this simulation represent at least metastable states.

(Page 5, line193) ... We assessed the stability of these representative structures in clusters 1 and 2 (clusters having the two largest populations) by running a simulation from representative structures. Four structures were sampled from cluster 1 and 2 each by randomly resampling structures with weight factors obtained from the reweighting. Then, 500 ns conventional MD simulations from these 4x2 structures were performed with new random initial velocities assigned. . ...

3. Combined with the problem with insufficient sampling in replica states, we revised the manuscript to inform readers the information and carefully stated the limitation of the current research:

(Page 6, line 226, points 1 and 3 combined) We first monitored the convergence of the ensemble using the secondary structure distribution and the stability of the hydrogen bonds between nsp1 and SL1 (Appendix A and Figs. SF2 and SF3 in S1 Text). The hydrogen bond and secondary structure statistics reached plateau at ~30 ns. However, as expected from the relatively short simulation length and large number of replicas, the replica states were not well mixed. ...

(Page 11, line 432) First, as we explained in subsection "Convergence of the extended ensemble simulation", even though the current simulation uses the extended ensemble method, it is difficult that the full convergence is achieved. ...

4. We revised the manuscript so that the manuscript is more comprehensive to readers outside the field of the molecular dynamics. Especially, we added a summary of the simulations performed at the beginning of "Materials and Methods" section as "Overview":

(Page 2, line 42) We constructed a complex of nsp1 and 5'-UTR of SARS-CoV-2 RNA and performed simulations to investigate the mechanism behind the self-evasion of the nsp1's translation shutoff. Nsp1 has an intrinsically disordered region (IDR) and is considered to bind to the RNA. However, it has generally been considered that RNA-protein complexes are difficult to simulate because structures tend to be trapped around the initial configurations in a reasonable simulation time, due strong charge-charge interactions between the RNA and the positively charged protein residues. To ease the problem, we performed an extended ensemble simulation. ...

In response to *"Actually, could you please explain better which points exactly are used for clustering, what exactly is in the matrix?"*, we added the explanation why we used binary matrix for the clustering:

> *(Page 5, line 163) After we obtained multiple poses of the nsp1-SL1 complex from the simulation, we classified structures into clusters. Typically, measures such as the root-mean-square distance (RMSD) are used to distinguish different structures; however, for the IDR, the RMSD is not informative because the structures are more diverse, and also because the RMSD is extremely sensitive to motions far from the center of mass. We thus used the contact information between nsp1 and SL1 residues to analyze the simulation results. …*

We believe these revisions made the manuscript clearer.

> *Interpretation of the paragraph "Distance between the nsp1 N-terminal…..". Is it not possible that the short distance in the MD ensemble (if I understood the text correctly) is merely due to over-compaction of the protein by the force field? Unless simulations of the free protein are made for comparison, it is difficult to judge, to my opinion. Here even standard simulation of the protein can help (considering the timescale of the REST2 it looks that the effect occurs fast).*

As we commented in the response to the reviewer 1, we performed additional simulations and removed this paragraph.

> *Have the authors made all data and (if applicable) computational code underlying the findings in their manuscript fully available?*
>
> *Reviewer #1: Yes*
>
> *Reviewer #2: None*

Upon submitting the original manuscript version, we already deposited the input structure as well as topology files and output trajectories to the model structure archive (BSMA, https://bsma.pdbj.org/entry/26), and we uploaded source code modifications to the SI data. However, it was not clear what were deposited on BSMA in the previous manuscript. We now explicitly state in the manuscript which data is deposited on BSMA. We believe that the deposited data are sufficient to reproduce this research.

We thank again for reviewers and editors processing this manuscript. With revisions made in this version, we believe our manuscript is suitable for publication in PLoS CB.
