# Supplementary material for: Extended ensemble simulations of a SARS-CoV-2 nsp1–5’-UTR complex
Source: PLoS Comput Biol. 2022 Jan 19;18(1):e1009804. doi: 10.1371/journal.pcbi.1009804 (PMC8803185; doi:10.1371/journal.pcbi.1009804)
Supplement: Attachment — Submitted filename: response.pdf [file pcbi.1009804.s005.pdf]

We thank again both reviewers for carefully reading the revised manuscript. Our responses to the individual comments are presented below.

> *Reviewer #2: The revision has been done more or less appropriately, although the convergence issue is bigger than expected/admitted by the authors. On the other hand, amount of simulations is reasonably large in the context of contemporary literature and, mainly, the system is overwhelmingly complex. Thus, from my point of view the paper is OK for publication. (Obviously, I cannot speak for the other Reviewer who commented on several problems that are outside my expertise and which I cannot competently judge.) Still, I would tend to suggest as minor revision to add very few references at places where the authors discuss the limitations, which would (without any further explanations) guide interested readers to the literature where a more thorough discussion can be found.*
>
> *p. 6. "However, as expected from the relatively short simulation length and large number of replicas, the replica states were not well mixed. The replica state indices of each continuous trajectory were limited in a narrow range, showing a sign of insufficient sampling (Fig. SF4 in S1 Text)".*
> *It obviously is not a sign of insufficient sampling but a clear demonstration of it. However, with the present system one can hardly do more. Still, some readers might be unaware what is the significance/meaning of not being well mixed and further, might be unaware that even good mixing would not guarantee a convergence. There are other issues such as the lack of decorrelation, which may be problem even in case of at first sight good replica mixing in RE (the replicas can travel well across the ladder and still there could be lack of decorrelation). Simple cross-referencing without further explanations to e.g., the Zuckerman's review https://www.annualreviews.org/doi/10.1146/annurev-biophys-042910-155255 plus the recent work https://www.nature.com/articles/s41467-021-21105-7 could help the readers. The former paper quite well explains the requirements for convergence: effective sample size, initial equilibration vs. correlation time etc. The latter showing system-dependent difficulties of specifically REST2.*
>
> *The Figure SF4 is useful.*

We revised the manuscript to explain the limitation and the problem of the present simulation. We added Zuckerman's review to explain the limitation of the current simulation:

> *Current simulation results should thus be considered to achieve only partial convergence at best, i.e., current structures may not be the fully determined*

most stable structure under the current simulation force field, nor may it encounter enough transitions to obtain unbiased samples.[60]

In fact, in the previous revision, we considered but decided not to add the citation to REHT method (Appadurai 2021 Nature comm. paper) because the method cannot be used for the present system as the globular part of nsp1 may unfold, and more importantly, the problems in our simulation research were mainly caused by our simulation setup rather than caveats in the REST2 protocol. Thus, we added the literature in the future research section:

... Additionally, recent researches suggest possible caveats and remedies in the REST2 protocol;[68,69] the combination of methodological advances and more refined models may enable us to sample structures such that the stability of the complex can be discussed quantitatively....

Similarly on p. 11 somewhere around line 460 probably a general reference to prot-RNA systems might be useful, e.g., https://pubs.acs.org/doi/10.1021/acs.chemrev.7b00427. There are specific layers of problems when simulating protein-RNA interactions compared to just RNA or protein simulations.

We thank the reviewer for the suggestion. We added the citation to the corresponding part of the manuscript.

... Not only force fields for proteins, but the choices for RNA force fields should also be considered, as each force field has different characteristics upon reproducing RNA structures as well as protein-RNA complexes [57,58,67]. The simulations with multiple different force fields will be almost necessary to avoid drawing conclusions biased by a specific force-field....
